# Miltefosine and Benznidazole Combination Improve Anti-*Trypanosoma cruzi In Vitro* and *In Vivo* Efficacy

**DOI:** 10.3389/fcimb.2022.855119

**Published:** 2022-07-05

**Authors:** Julián Ernesto Nicolás Gulin, Margarita María Catalina Bisio, Daniela Rocco, Jaime Altcheh, María Elisa Solana, Facundo García-Bournissen

**Affiliations:** ^1^ Instituto Multidisciplinario de Investigaciones en Patologías Pediátricas (IMIPP), Consejo Nacional de Investigaciones Científicas y Tecnológicas (CONICET)-Gobierno de la Ciudad de Buenos Aires (GCBA), Servicio de Parasitología y Enfermedad de Chagas, Hospital de Niños “Dr. Ricardo Gutiérrez, Ministerio de Salud, Buenos Aires, Argentina; ^2^ Instituto de Investigaciones Biomédicas (INBIOMED), Facultad de Medicina Universidad de Buenos Aires (UBA) – CONICET, Buenos Aires, Argentina; ^3^ Instituto Nacional de Parasitología (INP) ‘Dr. Mario Fatala Chaben’-Administración Nacional de Laboratorios e Institutos de Salud (ANLIS) ‘Dr. Carlos G. Malbrán’, CONICET, Buenos Aires, Argentina; ^4^ Instituto de Microbiología y Parasitología Médica (IMPaM), Universidad de Buenos Aires, Buenos Aires, Argentina; ^5^ Departamento de Ciencias Básicas, Universidad Nacional de Luján, Buenos Aires, Argentina; ^6^ Division of Pediatric Clinical Pharmacology, Department of Pediatrics, Schulich School of Medicine & Dentistry, University of Western Ontario, London, ON, Canada

**Keywords:** Chagas disease, *Trypanosoma cruzi*, miltefosine, benznidazole, drug combination chemotherapy, drug repositioning

## Abstract

Drug repurposing and combination therapy have been proposed as cost-effective strategies to improve Chagas disease treatment. Miltefosine (MLT), a synthetic alkylphospholipid initially developed for breast cancer and repositioned for leishmaniasis, is a promising candidate against *Trypanosoma cruzi* infection. This study evaluates the efficacy of MLT as a monodrug and combined with benznidazole (BZ) in both *in vitro* and *in vivo* models of infection with *T. cruzi* (VD strain, DTU TcVI). MLT exhibited *in vitro* activity on amastigotes and trypomastigotes with values of IC_50 =_ 0.51 µM (0.48 µM; 0,55 µM) and LC_50 =_ 31.17 µM (29.56 µM; 32.87 µM), respectively. Drug interaction was studied with the fixed-ration method. The sum of the fractional inhibitory concentrations (ΣFICs) resulted in ∑FIC= 0.45 for trypomastigotes and ∑FIC= 0.71 for amastigotes, suggesting *in vitro* synergistic and additive effects, respectively. No cytotoxic effects on host cells were observed. MLT efficacy was also evaluated in a murine model of acute infection alone or combined with BZ. Treatment was well tolerated with few adverse effects, and all treated animals displayed significantly lower mean peak parasitemia and mortality than infected non-treated controls (p<0.05). The *in vivo* studies showed that MLT led to a dose-dependent parasitostatic effect as monotherapy which could be improved by combining with BZ, preventing parasitemia rebound after a stringent immunosuppression protocol. These results support MLT activity in clinically relevant stages from *T. cruzi*, and it is the first report of positive interaction with BZ, providing further support for evaluating combined schemes using MLT and exploring synthetic alkylphospholipids as drug candidates.

## Introduction

Chagas disease, caused by the hemoflagellate *Trypanosoma cruzi*, is considered a neglected tropical disease due to the scarcity of safe and effective treatments ^1^. Current therapeutic options are limited to nifurtimox (NFX) and benznidazole (BZ), both developed over 40 years ago.

Etiological treatment during the acute stage results in high rates of parasitemia clearance and serological negativity, while treatment success during the chronic phase is currently arguable. Adverse events are usual, especially in adults, forcing treatment discontinuation (Pérez-Molina and Molina, 2018).

Despite the urgent need to develop safer drugs, the pharmaceutical industry has shown limited interest in research for new chemical entities, probably due to the socioeconomic condition of most affected patients (Paulino et al., 2005).

Drug repurposing (*i.e.*, the study of drugs already developed and tested for other diseases) (Chatelain and Ioset, 2011) and combination therapy arise as cost-effective strategies to fill the gap in the drug discovery pipeline (Keenan et al., 2013a; Andrews et al., 2014). Additionally, a positive drug combination increases drug efficacy by targeting multiple metabolic pathways, minimizing the risk of drug resistance, and reducing the frequency and intensity of dose-dependent adverse reactions (Field et al., 2017).

Initially developed for treating skin metastases in breast tumors, miltefosine (MLT), also known as hexadecylphosphocholine, is the first and to date only oral drug for visceral and cutaneous leishmaniasis (Dorlo et al., 2012). Extensive experience with MLT in leishmaniasis clinical trials suggests an excellent safety profile for patients, including pediatric patients, with low to moderate adverse events (Singh et al., 2006; Mbui et al., 2019). Still, some concerns remain regarding MLT effects on the gastrointestinal tract and potential teratogenicity (Dorlo et al., 2012).

MLT is considered a protein kinase B (PKB) inhibitor with a key role in intracellular signaling for cell viability (Dorlo et al., 2012). However, the mechanisms of action related to its anti-protozoal activity are not fully clarified yet. Some evidence suggests an inhibitory effect on phosphatidylcholine and sphingomyelin synthesis, triggering apoptosis (Maya et al., 2007; Apt, 2010) or inhibiting the mitochondrial cytochrome c oxidase and altering the organelle membrane (Dorlo et al., 2012; Ghosh et al., 2013). Similarly, it was proposed that MLT inhibits the parasite phosphatidylcholine biosynthesis through the transmethylation pathway (Lira et al., 2001).

Recently, it was proposed that MLT targeted the sphingosine-activated plasma membrane directly Ca^+2^ channel, both on *Leishmania donovani* and *T. cruzi*, affecting the intracellular calcium homeostasis (Benaim et al., 2020).

Previous reports indicated that MLT exhibits both *in vitro* (Croft et al., 1996; Santa-Rita et al., 2000) and *in vivo* activity (Saraiva et al., 2002; Martinez-Peinado et al., 2021) against *T. cruzi.*

However, *in vivo* efficacy was evaluated by different monotherapy treatment schemes and cure criteria hampering the assessment of the MLT ability to eradicate *T. cruzi* infection. Therefore, we conducted a set of *in vitro* and *in vivo* experiments based on previous guidelines (Romanha et al., 2010) to evaluate MLT efficacy alone and in combination with BZ.

## Materials and Methods

### *In Vitro* Studies

*Cell culture*

Vero-C76 cells (obtained from Asociación Banco Argentino de Células, an ATCC-certified cell bank) were cultured in T25 or T75 flasks with RPMI-1640 medium (Life Technologies Corporation, Grand Island, New York, USA) at 37°C in an atmosphere of 5% CO_2_, supplemented with 2.5 g/L sodium bicarbonate and 5% fetal bovine serum (FBS) (Natocor, Córdoba, Argentina) and penicillin (100 UI/mL) - streptomycin (50 μg/mL) or gentamicin (25 μg/mL).

### Parasite Strain

The VD strain of *T. cruzi* (DTU TcVI) was previously characterized (Gulin et al., 2018), and it was used throughout these studies. Bloodstream trypomastigotes were obtained from infected CF-1 outbred mice at parasitemia peak, isolated as previously described (Brossas et al., 2017), and maintained in cell culture for *in vitro* assays or used directly for *in vivo* studies.

### Trypanocidal Activity

The trypanocidal activity was assayed on cell culture-derived trypomastigotes. Experiments were carried out in 96-well microplates containing 10^6^ parasites/mL, in 100 µL as final volume. Serially diluted (160-0.156 µM) pure drugs (MLT, BZ, and NFX) were added to the wells, and the plates were incubated at 37°C for 24 h. After incubation, reduction in *T. cruzi* viability was determined by counting motile trypomastigotes in Kova International, Inc., Garden Grove, CA, USA. Each drug concentration was evaluated in triplicate, and control cultures were maintained without the drug. The lytic concentration 50% (LC_50_), defined as the drug concentration that resulted in a 50% reduction compared to the untreated control, was estimated by non-linear regression analysis plotting the percentage of viable trypomastigotes against the log of drug concentration (Rodrigues et al., 2014).

### Inhibition of Trypomastigotes Egress Test

Egress of trypomastigote forms from host cells was used as an indirect method to assess the arrest of amastigote development. Vero C-76 cells, plated at a density of 10^4^ cells/well in 96-well plates, were infected with 10^6^ parasites/mL and incubated at 37°C and 5% CO_2_ for 24 h. The wells were washed with PBS 1X to remove non-attached parasites, and the infected cell cultures were treated with serially diluted (160-0.156 µM) pure drugs (MLT, BZ, NFX) by triplicate. Control cell cultures were maintained without treatment. Trypomastigotes’ egress from host cells was evaluated on the 5^th^ day post-infection (dpi) as previously described (Polanco-Hernández et al., 2012). The number of parasites egressed was determined by counting viable trypomastigotes in the supernatant using the KOVA Glasstic slides^®^. The inhibitory concentration 50% (IC_50_), defined as the drug concentration resulting in a 50% reduction of trypomastigotes’ egress from infected cells, was estimated by non-linear regression analysis plotting the percentage of viable trypomastigotes against the log of drug concentration (da Silva et al., 2010; Polanco-Hernández et al., 2012).

### Determination of Cytotoxicity

Vero C-76 cells were plated at a density of 10^4^ cells in 96-wells plates and maintained at 37°C in an atmosphere of 5% CO_2_ overnight. Then, they were incubated in the presence of the compounds in serially diluted concentrations (160-0.156 µM). Each drug concentration was evaluated in triplicate, and control cultures were maintained without the drug. After 5 days, plates were washed and 100 μL of RPMI-5% SFB with 10% of a 3 mM resazurin solution (Sigma Aldrich, Saint Louis, MO, USA), were added to each well. The plate was incubated at 37°C with a 5% CO_2_ atmosphere for 4 h, and the reduction of resazurin was read in an ELISA reader (Thermo Fisher Scientific Oy, Vantaa, Finland) at 570-595 nm. Optical densities were analyzed to obtain the dose-response curve and the cytotoxic concentration 50 (CC_50_), defined as the concentration of compound that reduced the cell viability by 50% compared to untreated controls. Finally, the selectivity index (SI) was calculated as the CC_50_ for Vero cells/IC_50_ for *T. cruzi* parasites (Miranda et al., 2015).

### Drug Combination Assays

*In vitro* tests were carried out to identify the LC_50_ and IC_50_ of each drug separately (see above). For combination studies, dilutions were made by the fixed-ratio method, where the LC_50_ or the IC_50_ of one of the drugs remained constant, and the other drug was diluted in fixed ratios of the obtained LC_50_ or IC_50_ (Fivelman et al., 2004).

To assess trypanocidal activity, tested MLT concentrations (30.85; 7.71; 1.93 µM) were added to the pre-determined LC_50_ value of BZ at a fixed concentration. Similarly, the MLT LC_50_ value was added to serially diluted BZ (9.43; 2.36; 0.59 µM).

To study the inhibitory amastigote effect, selected MLT concentrations (0.51; 0.25; 0.12; 0.06; 0.03) were added to the pre-determined BZ IC_50_ value at fixed concentration. Similarly, the MLT IC_50_ was added to serial dilutions of BZ (0.72; 0.36; 0.18; 0.09; 0.04 µM).

For both cases, the Fractional Inhibitory Concentration Index (FICI) was calculated according to Cantin and Chamberland (1993) where,

LC_50_ or IC_50_ of A in combination LC_50_ or IC_50_ of B in combination


$$\sum \text{FICI}=\frac{{\text{LC}}_{50}\;{\text{IC}}_{50}\;\text{of}\;\text{A}\text{in}\;\text{combination}}{{\text{LC}}_{50}\;or\;{\text{IC}}_{50}\;\text{A}\;\text{alone}}\;+\;\frac{{\text{LC}}_{50}\;{\text{IC}}_{50}\;\text{of}\;\text{B}\text{in}\;\text{combination}}{{\text{LC}}_{50}\;\text{or}\;{\text{IC}}_{50}\;\text{B}\;\text{alone}}$$

LC_50_ or IC_50_ of A alone LC_50_ or IC_50_ of B alone

FICI values were interpreted as follows: ≤ 0.5, synergistic; 0.5 > 4, additive and ≥ 4 antagonistic interaction (Odds, 2003).

### *In Vivo* Assays

#### Experimental Animals

Twenty-one-day-old female BALB/cJ mice (16 ± 2 grams) were purchased from the Faculty of Veterinary Sciences, University of Buenos Aires, Argentina, and housed under conventional closed barriers at Ricardo Gutiérrez Children’s Hospital animal facilities (Buenos Aires, Argentina).

Animals were kept at five mice per cage in 600 cm^2^ polycarbonate cages, including plastic tubes as environmental enrichment and nesting material. Animals were individually identified, and cages were properly labeled. Cages were filled with irradiated chip-bedding, which was changed once a week. Mice had access to food (Rata-Ratón Cooperación^®^, Buenos Aires, Argentina) and filtered water *ad libitum*. Macroenvironmental conditions included a 12:12-h light: dark cycle (starting at 6 a.m.), controlled temperature (20 ± 2°C), and humidity (55 ± 10%).

Procedures for housing and handling animals followed international guidelines for animal care and welfare (National Research Council, 2011). The study protocol was approved by the Institutional Animal Care and Use Committee from the Faculty of Veterinary Sciences (University of Buenos Aires) (Protocol #2014/04).

### Infection and Treatment Schedules

Five-week-old mice were infected by intraperitoneal (ip) injection with 500 bloodstream trypomastigotes from the VD strain of *T. cruzi*. After the infection was established, mice with patent parasitemia were assigned by simply randomization to treatment groups as indicated in **Table 1** (monotherapy) and **Table 2** (combinatory therapy).

**Table 1:** Effect of miltefosine, benznidazole, or nifurtimox on parasitemia parameters in BALB/cJ mice infected with *Trypanosoma cruzi* (VD strain) ^a^.

Treatment(mg/kg/day)	n	PPP (days)	MPR (trypomastigotes/mL)	MPRreduction(%)	dMPR (median (range))	Parasitemia at end of treatment (28 dpi) (trypomastigotes/mL)	Survival (%)
MLT (25)	5	35.00 (9.80)^D^	1.03x10^6^ (± 0.24x10^6^)^A^	50.12	19 (15- 21)^C^	1.56x10^4^ ( ± 3.13x10^4^)^A^	4/5 (80)^A^
MLT (50)	5	26.00 (2.74)^CD^	2.20x10^5^ (± 0.53 x10^5^)^B^	89.29	9 (9- 9)^AB^	2.25x10^4^ ( ± 1.63x10^4^)^B^	5/5 (100)^A^
MLT (75)	5	24.20 (3.83)^CD^	2.42x10^5^ (± 0.61x10^5^)^B^	88.20	9 (9- 9)^A^	2.00x10^4^ ( ± 1,4.3x10^4^)^B^	5/5 (100)^A^
MLT (100)	7	15.29 (4.07)^BC^	7.68x10^4^ (± 6.43 x10^4^)^B^	96.26	12 (12- 15)^BC^	0.00 ( ± 0.00)^C^	7/7 (100) ^A^
BZ (100)	9	3.88 (2.23)^AB^	1.46x10^5^ (± 1.15x10^5^)^B^	92.02	11 (9- 12)^AB^	0.00 ( ± 0,00)^C^	9/9 (100)^A^
NFX (100)	10	4.00 (4.32)^A^	8.25x10^4^ (± 7.66 x10^4^)^B^	95.99	9 (9- 9)^A^	0.00 ( ± 0.00)^C^	10/10 (100)^A^
NT (—)	14	—	2.06x10^6^ (± 0.91 x10^6^)^A^	—	17 (17- 21)^C^	—	0/14 (0)^B^

MLT, miltefosine. BZ, benznidazole. NFX, nifurtimox. NT, infected non-treated. PPP, patent parasitemia period. MPR, maximum parasitemia reached; dMPR, day of maximum parasitemia reached.

Female BALB/cJ mice were infected with 500 trypomastigotes of T. cruzi VD strain; treatment started at 8 dpi and was administered orally for 20 consecutive days.

Different letters indicate significant differences (Kruskal-Wallis; p < 0.05). Values are expressed as mean (± standard deviation) except where indicated.

**Table 2:** Effect of miltefosine alone or combined with benznidazole on parasitemia parameters in BALB/cJ mice infected with *Trypanosoma cruzi* (VD strain).

Treatment(mg/kg/day)	n	MPR(trypomastigotes/mL)	MPRreduction (%)	Survival(%)
Subtherapeutic regime				
MLT (25)	4	9.06x10^4^ ( ± 4.13x10^4^)^A^	76.23^A^	4/4 (100)^A^
BZ (5)	4	7.8x1x10^4^ ( ± 3.13x10^4^)^A^	79.51^A^	4/4 (100)^A^
MLT (25) + BZ (5)	4	7.81x10^4^ ( ± 4.49x10^4^)^A^	79.51^A^	4/4 (100)^A^
NT (—)	4	3.81x10^5^ ( ± 1.64x10^5^)^B^	—–	2/4 (50)^B^
Additive regime*				
BZ (100)	8	2.17x10^4^ ( ± 1.30x10^4^)^A^	94.31^A^	8/8 (100)^A^
MLT (50) + BZ (50)	8	3.63x10^4^ ( ± 1.24x10^4^)^A^	90.49^A^	8/8 (100)^A^
NT	12	3.81x10^5^ ( ± 1.64x10^5^)^B^	—	0/12 (0)^B^

NT, infected non-treated. MLT, miltefosine. BZ, benznidazole. MPR, Maximum parasitemia reached.

Female BALB/cJ mice were infected with 500 trypomastigotes of T. cruzi VD strain; treatment started at 8 dpi and was administered orally for 20 consecutive days. * Results obtained from two independent assays.

Different letters indicate significant differences (Kruskal-Wallis; p < 0.05). Values are expressed as mean (± standard deviation).

MLT (pure product provided by Laboratorio Dr. Lazar & Cía. S.A.Q. e I, Buenos Aires, Argentina) was prepared in a 100 mg/mL work solution and dissolved in sterile distilled water. BZ and NFX (Radanil, Roche, Buenos Aires, Argentina and Lampit, Bayer, Buenos Aires, Argentina) were used as reference treatments in this study. Commercial tablets were crushed and suspended in 0.25% carboxymethylcellulose solution (Sigma Aldrich, Saint Louis, MO, USA). To avoid potential pharmaceutical interactions between drugs, BZ was administered at least an hour before MLT in the combination treatment.

Treatment started immediately upon onset of parasitemia in all animals and it was administered daily for 20 consecutive days by oral route in 50 µL as final volume. Doses, length of treatment, and route of administration for monotherapy schemes were chosen based on published data (Saraiva et al., 2002; Romanha et al., 2010). For combinatory treatments, doses were carefully chosen to obtain sub-therapeutic (i.e., doses that are administered separately do not lead to parasitemia suppression) (Gulin et al., 2020) or additive effects (i.e., doses that are administered together add up to the therapeutic dose that leads to parasitemia suppression).

### Evaluation of Treatment Response

Clinical condition, including physical appearance and behavior based on previously established parameters (Olfert and Godson, 2000), as well as body weight and temperature, were recorded weekly.

Blood parasitemia was evaluated twice a week with a hemocytometer, a technique with a detection limit of 12,500 parasites/mL. Briefly, 5 μL of blood obtained from the vein tail was diluted 1:5 in lysis buffer (Tris-NH_4_Cl 0.83%, pH=7.2), and parasites were counted in a Neubauer chamber at 400X magnification.

Additional parameters were calculated to assess drug efficacy: patent parasitemia period (PPP), defined as the number of days in which parasitemia can be detected by direct observation; the maximum parasitemia reached (MPR), considered as the maximum parasitemia value achieved in each infected animal; and day of MPR (dMPR). Mortality was registered daily. Pre-established anticipated endpoints were used to avoid unnecessary pain and stress, and animals were euthanized if they fulfilled any of these criteria (i.e., 20% weight loss from initial body weight, body temperature lower than 33.5°C, or parasitemia ≥2x10^6^ trypomastigotes/mL). Euthanasia was performed with CO_2_ inhalation in a saturated chamber or with sodium pentothal overdose (300 mg/kg, ip).

### Cyclophosphamide-Induced Immune Suppression and Assessment of Cure

After the treatment schemes were completed, mice with negative parasitemia were submitted to an immunosuppression protocol with cyclophosphamide (CYP). CYP (Filaxis^®^; Martínez, Buenos Aires, Argentina) was diluted in sterile sodium chloride 0.45% and administered by ip route at 200 mg/kg once a week for 28 days. During the immunosuppression cycle, the presence of blood trypomastigotes was assessed periodically. If parasitemia did not rebound, animals were euthanized and blood, heart, and skeletal muscle samples were collected for parasite DNA quantification by qPCR and histology evaluation.

### PCR Sampling and Preparation

Blood samples were obtained by submandibular vein puncture (Golde et al., 2005) and collected in sterile cryotubes with 1:3 guanidine-HCl 6M-EDTA 0.2M pH 8 buffer. Skeletal muscle from the rear legs and heart were obtained and rinsed with sterile distilled water before being collected in separate sterile cryotubes. Samples were stored at -20°C until processing.

DNA was extracted with High Pure PCR Template Preparation Kit (Roche Diagnostics GmbH, Mannheim, Germany) according to the manufacturer’s protocol. To discard inhibitions, an internal amplification control (0.2 ng) was included in each sample before starting the DNA extraction procedure (Duffy et al., 2013). Extracted DNA was quantified by spectrophotometry at 260 nm wavelength in a Nanodrop 1000 spectrophotometer (Thermo Fisher Scientific, Wilmington, DE, USA) and stored at -20°C until use.

### Quantitative PCR

Amplification was performed using oligonucleotides targeted to a 166 bp *T. cruzi* satellite DNA fragment and IAC on a StepOne PCR system (Applied Biosystems, Foster City, California, USA) as described elsewhere (Duffy et al., 2013). qPCR conditions and standard parasite curve for data analysis were performed as previously described (Gulin et al., 2018). The efficiency of amplification was determined using the following calculation: Efficiency (E) = 10^(−1/slope)^, using Step One Software 2.1 (Applied Biosystems, Foster City, California, USA).

Positive and negative blood or tissue samples and reagent controls were run in each assay. DNA extraction, mixing, and qPCR reaction were performed in separate areas to avoid contamination. The cycle threshold (Ct) allowed interpolating the parasite equivalents of each sample from the standard curve.

### Tissue Samples Preparation and Histological Analysis

Segments of heart and skeletal muscle were fixed in buffered 10% formaldehyde, dehydrated, and embedded in paraffin. Then, 5 mm thick sections were stained with hematoxylin and eosin (H&E). A single-blind evaluation of the specimens was performed by light microscopy, and the parasite load was expressed as the presence or absence of amastigote nests. The degree of myocardial and skeletal muscle inflammation was scored as previously reported (Tarleton et al., 1994; Solana et al., 2012).

### Statistical Analysis

For *in vivo* studies, data distribution and homoscedasticity were evaluated. If the distribution was normal and variance was uniform, analysis of variance (ANOVA) and correction for multiple comparisons with Bonferroni test, when necessary, were performed. Data with distributions other than normal were analyzed by non-parametric Kruskal-Wallis test and compared in pairs. Survival analysis was performed using the Kaplan-Meier test.

In all cases, p-values <0.05 were considered statistically significant. Statistical analyses for *in vivo* tests were performed with InfoStat/P 2014 program, while analysis of the *in vitro* data and graphics were prepared with GraphPad Prism 5.03 (GraphPad Software, San Diego, California, USA). Values in tables and graphs are expressed in mean values with standard deviation unless otherwise indicated.

## Results

### *In Vitro* Anti-*T. cruzi* Activity of MLT Alone and Combined With BZ

The *in vitro* anti-*T. cruzi* activity and cytotoxicity of MLT, BZ, and NFX alone are summarized in **Table 3**. The MLT trypanolytic effect was significantly less potent than BZ or NFX, obtaining an LC_50_ value near the high micromolar range. However, MLT exhibited an *in vitro* amastigote inhibitory activity similar to BZ and NFX.

**Table 3:** *In vitro* activity of miltefosine, benznidazole, and nifurtimox on trypomastigote and intracellular amastigote stages of *Trypanosoma cruzi* (VD strain) and their cytotoxic effects on Vero cells.

Drug	LC_50_(Anti-trypomastigote activity)	IC_50_(Anti-amastigote activity)	CC_50_(Host-cell cytotoxic activity)	SI
**MLT**	31.17 (29.56; 32.87)	0.51 (0.48; 0,55)	57.36 (36.14; 91.05)	112
**BZ**	9.43 (8.62; 10.32)	0.73 (0.69; 0.77)	> 640	> 876
**NFX**	2.35 (2.00; 2.78)	0.15 (0.13; 0.17)	> 220	> 1.497

MLT, miltefosine. BZ, benznidazole. NFX, nifurtimox.

LC_50_: lytic concentration 50%; drug concentration needed to reduce the trypomastigote motility by 50% compared to the infected non-treated control.

IC_50:_ inhibitory concentration 50%; drug concentration needed to reduce the intracellular amastigote development by 50% compared to the infected non-treated control.

CC_50_, cell toxicity 50%; drug concentration capable of reducing the cell viability by 50% compared to the non-treated cell culture.

SI, selectivity index. SI, CC_50_/IC_50_. Values are expressed in µM and reported as the mean concentration and the 95% confidence interval (IC95%).

**Figure 1:**
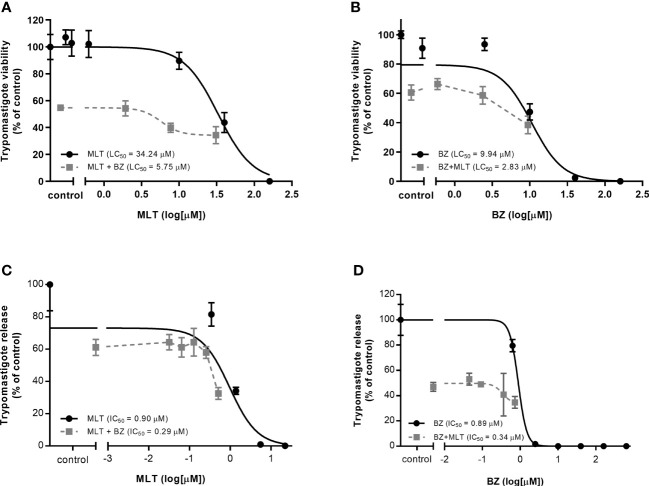
*In vitro* activity of miltefosine (MLT) and benznidazole (BZ) combination on **(A-B)** trypomastigotes and intracellular amastigote development **(C-D)** of *Trypanosoma cruzi* (VD strain). **(A)** LC_50_ value from BZ combined with variable concentrations of MLT.**(B)** LC_50_ value from MLT combined with variable concentrations of BZ. **(C)** IC_50_ value from BZ combined with variable concentrations of MLT. **(D)** IC_50_ value from MLT combined with variable concentrations of BZ. Values are expressed in µM and reported as the mean concentration and the 95% confidence interval (IC95%).

The *in vitro* activity of MLT and BZ combination on trypomastigotes and amastigotes stages of *T. cruzi* is summarized in **Table 4**, and isobolograms are shown in **Figure 1**. The MLT and BZ association led to a significant decrease in trypomastigotes viability, assessed as a drop in the LC_50_ value. Moreover, the ΣFICI suggested that this combination would have a synergic effect on trypomastigotes lysis in culture. Similarly, the *in vitro* MLT and BZ effect on the amastigote stage reduced the IC_50_ value compared to the IC_50_ of each drug separately. Thus, the ΣFICI suggested an additive interaction.

**Table 4:** *In vitro* fractional inhibitory concentration index from miltefosine and benznidazole on trypomastigotes and amastigotes stages of *Trypanosoma cruzi* (VD strain).

Combination	Trypomastigotes			Amastigotes		
	LC_50_ (µM)	FICI	∑FICI	IC_50_ (µM)	FICI	∑FICI
**Fixed MLT +** **Variable BZ**	2.845	0.17	0.46	0.34	0.38	0.71
**Fixed BZ +** **Variable MLT**	5.75	0.29		0.29	0.32	

MLT, miltefosine. BZ, benznidazole. FICI, Fractional inhibitory concentration index.

### *In Vivo* Evaluation of MLT as Monotherapy

#### Clinical Response

Mice treated with MLT exhibited slight signs of discomfort during oral administration, denoted by retching, anxious appearance, and repeated grooming of lips and cheeks immediately after treatment. These signs lasted 15-20 minutes and then the animals returned to their usual behavior. These changes were not observed in the non-treated infected group administered the same volume of vehicle (i.e., distilled water or CMC 0.25%), nor in the mice infected and treated with BZ or NFX.

Throughout MLT treatment, the mice experienced a decrease in body weight, although these differences were not statistically significant compared with the other experimental groups (**Supplementary Material**, **Figure S1.A**). In the groups treated with MLT at 50 or 75 mg/kg/day, the bodyweight remained between the initial and an average increase of 20%, while the mice treated with 100 mg/kg/day of MLT showed a marked weight decrease from 11 dpi, but they recovered at 21 dpi and increased by an average of 9.4% compared to the initial weight by the end of the treatment (28 dpi).

Body temperatures remained close to the lower limit of the normal range for the murine species, without significant differences between the treatment groups, possibly due to the wide dispersion of the obtained values (**Supplementary Material**, **Figure S1.B**).

However, the NT group showed a significant decrease in body temperature as the acute course of the infection progressed, which was accompanied by a reduction in body weight, lethargic behavior, and ruffled coat.

#### Treatment Response During the Acute Phase

**Figure 2:**
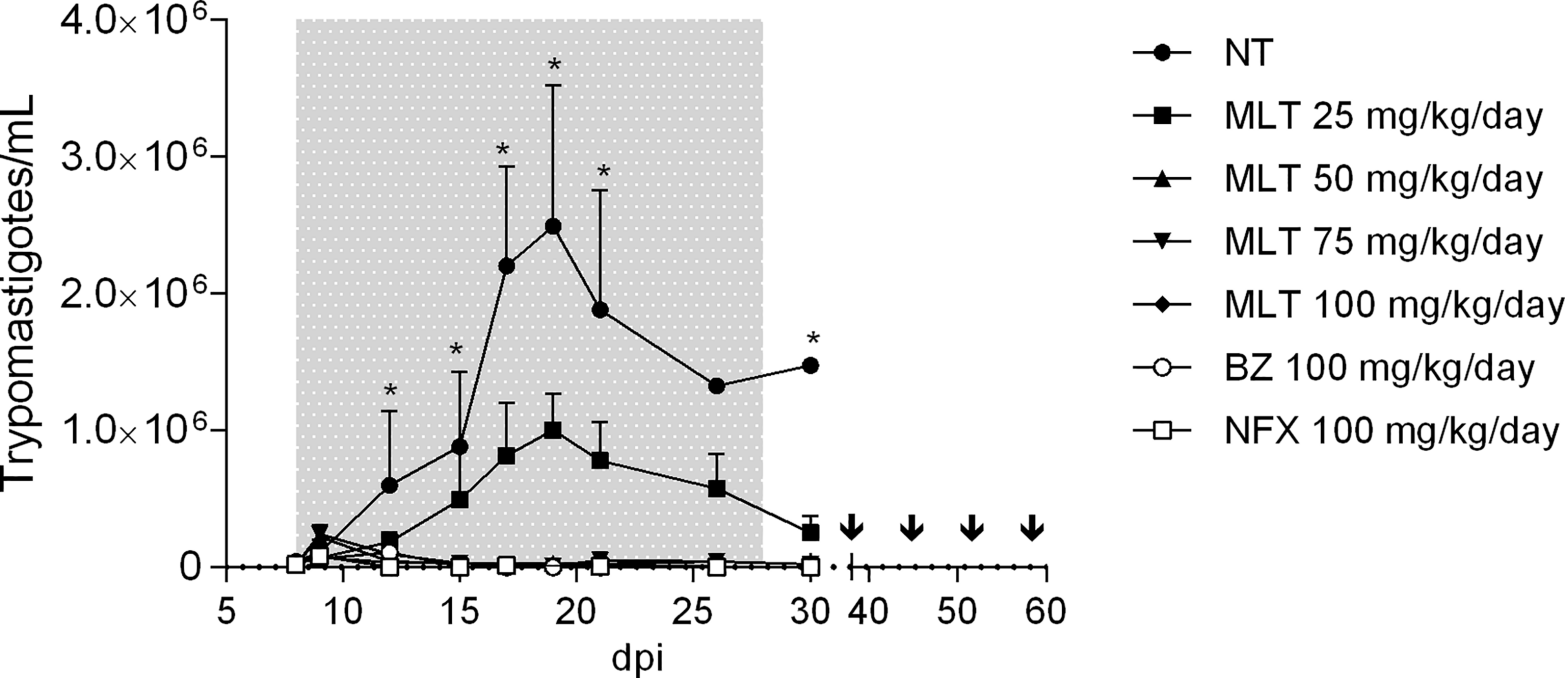
Effect of miltefosine, benznidazole, or nifurtimox on parasitemia course in BALB/cJ mice infected with *Trypanosoma cruzi* (VD strain). Values are expressed as mean trypomastigotes/mL (± SD) in peripheral blood from experimental groups according to the days after infection (dpi). Mice were inoculated with 500 trypomastigotes of the VD strain of *Trypanosoma cruzi*, and treatment started at parasitemia onset (8th dpi). During treatment and up to 10 days post-treatment, parasitemia was evaluated by fresh blood examination (FBE) to determine parasitemia rebound. Animals with negative parasitemia were submitted to immunosuppression consisting of four doses of cyclophosphamide (CYP; 200 mg/kg; ip route), separated by one week. Parasitemia was evaluated during the CYP cycle and up to 7 days after the last dose. The asterisks indicate significant differences compared to NT and MLT 25 mg/kg/day groups (Kruskal-Wallis, p < 0.05). Grey shading indicates the treatment period. Arrows indicate CYP administration cycle. NT, infected non-treated. MLT, miltefosine. BZ, benznidazole. NFX, nifurtimox.

MLT treatment led to suppressive effects on parasitemia and associated parameters in a dose-dependent manner (**Figure 2** and **Table 1**). The patent parasitemia period (PPP) was statistically different between the treatment groups (p <0.0001). MLT administered at 100 mg/kg/day significantly reduced the PPP compared to MLT at 25 mg/kg/day. In contrast, groups treated with MLT or BZ at 100 mg/kg/day did not lead to significant differences in PPP during the acute phase of infection.

Moreover, treatment with MLT in doses ≥50 mg/kg/day reduced maximum parasitemia reached (MPR) without significant differences compared to the reduction achieved with BZ or NFX. Animals treated with 100 mg/kg/day of BZ, NFX, or MLT had undetectable blood trypomastigotes at the end of the treatment (**Figure 2**).

Administration of MLT at doses ≥50 mg/kg/day prevented mortality in 100% of the mice. Groups treated with MLT at any dose, BZ or NFX, were statistically more likely to survive than NT controls (p<0.0001 for log-rank Mantel-Cox test).

#### Assessment of Parasitological Cure

At the end of the treatment, at least one mouse from groups receiving MLT at 25, 50, and 75 mg/kg/day exhibited patent parasitemia (1/4, 4/5, and 4/5, respectively). Conversely, all mice treated with 100 mg/kg/day of MLT, BZ, or NFX had negative parasitemia by optical microscopy. Through the following 10 days, parasitemia remained negative in all animals, regardless of the treatment previously administered.

The CYP immunosuppression cycle was initiated 10 days after treatment completion. The parasitemia rebound occurred throughout the follow-up period in all animals from MLT treated groups, usually between the second and fourth CYP dose.

Animals from BZ and NFX groups displayed parasitemia rebound in 44% (4/9) and 60% (6/10) of cases, respectively. qPCR on blood samples yielded the same positivity rate as microscopy.

#### Histopathology

**Figure 3:**
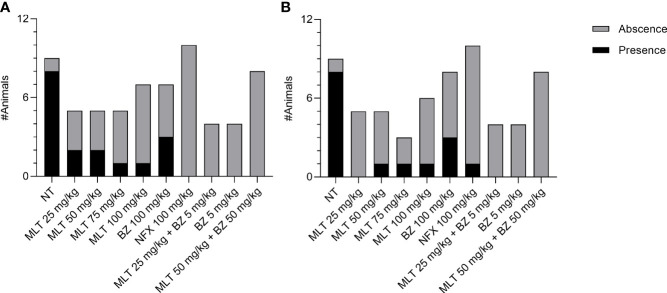
Effect of miltefosine (alone or in combined regimes), benznidazole, or nifurtimox on amastigote nest burden in BALB/cJ mice infected *Trypanosoma cruzi*, (VD strain). Mice with the presence or absence of amastigote nests in skeletal muscle **(A)** and heart samples **(B)**. NT, infected non-treated. MLT, miltefosine. BZ, benznidazole. NFX, nifurtimox.

Inflammation scoring in skeletal muscle samples was significantly different between treatment groups (p= 0.0025) (**Supplementary Material**, **Figure S2.A**). Inflammation in skeletal muscle was significantly lower in animals treated with MLT at 50 mg/kg/day than the NT group, similarly to animals treated with BZ or NFX. The other MLT doses studied had a wide range of variability in inflammation scores. Regarding the inflammation degree in the myocardium, there were no significant differences among treatment groups (**Supplementary Material**, **Figure S2.B**). The proportion of animals with amastigote nests was significantly higher in the NT group than in other treatment groups, in skeletal and myocardial muscle (Chi-square, p=0.0003 and 0.0044, respectively). No significant differences were detected between the groups receiving MLT, BZ, or NFX, both in skeletal muscle and myocardium (**Figure 3**). Microphotographs of representative skeletal and cardiac muscle samples from different treatment groups can be observed as **Supplementary Material** (**Figures S3**, **S4**, respectively).

### *In Vivo* Evaluation of MLT Combined With BZ

#### Clinical Response

Similar to the monotherapy trials, MLT administration in combined regimes produced a significant variation of the average weight gain from day 13 post-infection (6 days of treatment), with a decrease relative to the initial weight of between 10 and 15% in the animals treated with the MLT and BZ combination, which was then partially reversed toward the end of treatment. However, the initial weight was not recovered. Body temperature values were close to the lower limit of the normal range for the species recorded, without significant differences between the treatment groups (**Supplementary Material**, **Figures S5**, **S6**).

#### Treatment Response

During the acute course of the infection, treatments lead to a significant decrease in parasitemia. MLT and BZ administration in sub-therapeutic doses given separately or in combination diminished the MPR between 76.23 to 79.51%, which was significantly higher in the NT infected group compared to the treatment groups (p < 0.01) (**Table 2**). Likewise, administration of sub-therapeutic doses of MLT and BZ alone or in combination prevented mortality (**Supplementary Material**, **Figures S7**).

For the additive treatment regime, the combination of BZ+MLT at half the effective doses (i.e., 50 mg/kg/day each) significantly reduced MPR compared to NT animals, but there were no significant differences when compared with the group receiving BZ 100 mg/kg/day. Finally, 100% of the mice treated with MLT either alone or combined with BZ survived (**Supplementary Material, Figure S8**).

#### Assessment of Parasitological Cure

The treatment with sub-therapeutic doses of MLT or BZ, alone or in combination, was not able to eliminate circulating parasites and therefore the estimated parasitic load by qPCR was not significantly different (**Table 5**).

**Table 5:** Effect of miltefosine alone or combined with benznidazole at different sub-therapeutic or additive regimes on parasite burden at the end of the treatment in BALB/cJ mice infected with *Trypanosoma cruzi* (VD strain).

Treatment(mg/kg/day)	n	+FBE	qPCR					
			+Blood after CYP	Parasite Eq/mL blood	+BloodbeforeCYP	ParasiteEq/mL	+SkeletalMuscle	+CardiacMuscle
**Subtherapeutic regime**								
MLT (25)	4	4/4	4/4	4.099 ( ± 4.240)	ND	ND	ND	ND
BZ (5)	4	4/4	4/4	2.856 ( ± 2.275)	ND	ND	ND	ND
MLT (25) + BZ (5)	4	4/4	4/4	1.553 ( ± 2.154)	ND	ND	ND	ND
NT	1	1/1	1/1	163.734	ND	ND	ND	ND
Additive regime*								
BZ (100)	8	1/8	2/8 (2*)	NQ	3/8 (3*)	NQ	5/8 (1*)	4/8 (1*)
MLT (50) + BZ (50)	8	0/8	0/8	NQ	1/8 (1*)	NQ	4/8 (1*)	3/8 (2*)

MLT, miltefosine. BZ, benznidazole. NT, infected non-treated. CYP, cyclophosphamide (200 mg/kg; ip).

+FBE, positive parasitemia result in fresh blood examination at the end of the treatment.

+SkM, positive result for qPCR assay in skeletal muscle.

+CM, positive result for qPCR assay in cardiac muscle.

NQ, not quantifiable sample.

ND, not determined.

*Includes positive but not quantifiable samples.

Regarding the additive treatment regime, mice receiving BZ at 100 mg/kg/day or MLT+BZ at 50 mg/kg/day showed negative parasitemia by fresh blood examination after 20 consecutive days of treatment. The experimental groups were kept under observation from that moment and the following 10 days, and parasitemia remained negative in all subjects, regardless of the administered treatment.

After that period, the immunosuppression cycle started in all mice (CYP, 200 mg/kg/week, ip route). Mice initially treated with MLT+BZ exhibited negative parasitemia at the end of the immunosuppression cycle, while only one animal from group BZ (100 mg/kg/day) showed parasitemia rebound.

Later, blood qPCR showed that 3/8 animals treated with BZ 100 mg/kg/day presented detectable non-quantifiable DNA. Whereas only one mouse treated with MLT+BZ combination exhibited detectable but non-quantifiable parasitic DNA in blood after immunosuppression (**Table 5**). Parasitic DNA was detected in low quantity in skeletal and cardiac muscle samples, and it could not be quantified with the previously established curve. The proportion of positive samples in skeletal and cardiac muscle was not statistically significant between treatment groups (**Table 5**).

#### Histopathology

Regarding the combinatory therapy, inflammation scores in skeletal muscle and cardiac muscle were not significantly different among treatment groups. A scarce inflammatory infiltrate was observed, assigning scores ranging from 0 to 3 for skeletal muscle and between 0 and 2 for cardiac muscle, with no significant differences (**Supplementary Material**, **Figure S2.A**, **S2.B**). Moreover, amastigotes nests could not be recorded in any sample from treatment groups (**Figure 3**). Microphotographs of representative skeletal and cardiac muscle samples from different treatment groups can be observed as Supplementary Material (**Figure S3**, **S4**, respectively).

## Discussion

MLT is a lipophospholipid analog developed originally as an antineoplastic agent (Dorlo et al., 2012). Based on previous *in vitro* and *in vivo* studies and results from oncology clinical trials, MLT was repositioned for visceral and cutaneous leishmaniasis treatment. Since its registration in 2002, MLT remains the only oral drug available for leishmaniasis (Reimão et al., 2020).

MLT and other alkyl-lipophospholipids have proven activity on *T. cruzi* (Croft et al., 1996; Luna et al., 2009). Our results support the MLT effect on inhibiting amastigote development *in vitro* and the low efficacy on trypomastigote stage (Croft et al., 1996; Santa-Rita et al., 2000). Moreover, MLT high selectivity index suggests a specific antiparasitic mechanism of action without affecting the host cell.

To date, only three studies have assessed the *in vivo* efficacy of MLT (Croft et al., 1996; Saraiva et al., 2002; Martinez-Peinado et al., 2021) in mice models of acute infection. While Croft et al. observed transient parasitemia suppression without a curative effect or reduction of mortality in mice treated for 5 days at up to 30 mg/kg/day by oral route, Saraiva et al. reported parasitic sterilization in all mice treated with 25 mg/kg/day, determined by direct blood observation at 120 dpi and spleen culture for 15 days. Recently, Martinez-Peinado et al. studied MLT efficacy by giving 30 mg/kg/day for 10 days, using the Brazil strain expressing firefly luciferase, which yielded statistical differences in luciferase activity compared to infected non-treated mice.

It is well known that several experimental variables affect the infection course and the main outcomes in animal models of *T. cruzi* infection (Chatelain and Konar, 2015), and their heterogeneity make difficult the direct comparison between results.

In this work, we evaluated the *in vivo* efficacy of MLT using state-of-the-art methodology based on previous guidelines for drug screening (Romanha et al., 2010) by using qPCR and immunosuppression cycle to elucidate the possible reemergence of parasites from tissues.

The murine model of acute infection with *T. cruzi* was previously standardized (Gulin et al., 2018), using the VD strain (TcVI), originally isolated from a patient infected by a congenital route. This model becomes relevant since mother-to-child infection is the main transmission mode in vector-free areas within and outside Latin America (Carlier et al., 2019). Furthermore, the VD strain displays high parasitemia levels and mortality rates in infected non-treated mice and a moderate BZ and NFX effect to eradicate the infection.

Despite a temporary animal discomfort observed in MLT oral administration, the treatment was well tolerated by the animals. Also, the weight loss and ruffled fur agree with previously reported effects (Saraiva et al., 2002). The body weight loss could be explained by the gastrointestinal discomfort caused by MLT at all assayed doses, an adverse effect widely reported in clinical trials (Jha et al., 1999; Sundar et al., 2002).

As described by Saraiva et al., the parasitemia course throughout the acute phase of infection was not suppressed with 25 mg/kg/day of MLT. However, in our infection model, the days of patent parasitemia and the MPR were lowered in a dose-dependent manner by MLT, and the 100 mg/kg/day dose was able to reduce blood trypomastigotes circulation to undetectable levels by optical microscopy.

When administered alone, MLT at lower doses leads to a parasitemia reduction, and this could be explained in part due to the widespread drug distribution and the slow clearance rate. In mice, oral MLT is absorbed slowly but extensively and accumulates mainly in the kidneys, liver, and lungs (Breiser et al., 1987). Clinical pharmacokinetic studies indicate that, due to its prolonged half-life, MLT accumulates during treatment reaching a steady-state concentration in the last week of a 28-day treatment schedule (Kip et al., 2018).

Although the drug failed to produce parasitic sterilization, it was able to maintain the burden of amastigotes at low levels, which was reactivated by CYP-induced immunosuppression. Moreover, animals treated with MLT showed lower rates of amastigote nests in the myocardium and in skeletal muscle compared to NT, which could be explained by the MLT extensive distribution in these tissues, as previously documented in mice and rats (Breiser et al., 1987; Marschner et al., 1992).

Failure to reach parasitological sterilization was also detected in a percentage of the animals treated with BZ or NFX. These results coincide with the previously published characterization of the VD strain (Gulin et al., 2018), and with other *T. cruzi* strains from the same DTU, usually classified as BZ-sensitive (Fernández et al., 2010; Keenan et al., 2013b).

MLT effect on *Leishmania* spp. was also studied in combination with reference drugs or with repositioned compounds. *In vitro* additive synergism with sodium stibogluconate and *in vivo* potentiate synergism potentiation combined with paromomycin or amphotericin B were reported (Seifert and Croft, 2006). Costa et al. also reported the parasiticidal effect of efavirenz on *L. infantum* and a lower IC_50_ adding MLT (Costa et al., 2016).

The combined effect of MLT with other compounds on *T. cruzi* viability was previously described by *in vitro* combination with ketoconazole, both on trypomastigotes and intracellular amastigotes (Santa-Rita et al., 2005). However, this is the first study of the anti-*T cruzi* effect of the MLT and BZ combination.

The analysis of the MLT+BZ *in vitro* combination suggests a synergistic effect on trypomastigotes viability and an additive interaction on intracellular amastigote development. This association was not evaluated previously, although similar *in vitro* interactions were reported when combining MLT with paromomycin (de Morais-Texeira et al., 2014) or nitazoxanide (Mesquita et al., 2014) on *Leishmania* spp.

We furthered the evaluation of the MLT and BZ association on a murine model of acute infection with *T. cruzi.* MLT+BZ combination at sub-therapeutic doses could not achieve an effect different from that obtained with the drugs separately. However, it decreased parasite loads in blood and prevented mortality in treated mice. Nevertheless, MLT+BZ at half the total dose (i.e., 50mg/kg) produced negative parasitemia and prevented the reactivation after the immunosuppression protocol.

The qPCR yielded disparate results in target organs such as skeletal muscle and myocardium in both experimental groups, with detection of parasitic DNA in low-loading or even non-quantifiable cases. These results could then question qPCR as a method to establish parasitological cure in cases where *T. cruzi* DNA is not detected in the blood.

Residual DNA in tissues and phagocytic cells has been reported in experimentally infected mice treated with BZ and considered cured by different methods (Guarner et al., 2001; Martins et al., 2008), which would indicate the presence of non-viable parasites or even cross-contamination with parasitic DNA.

However, recently described spontaneous dormancy in *T. cruzi* (Sánchez-Valdéz et al., 2018) could also explain the presence of parasitic DNA in tissues due to the non-replicative amastigote stage in the absence of bloodstream trypomastigotes, which would deserve further investigation to fully understand therapeutic failure both in preclinical studies and clinical trials.

Although our results demonstrate the inability of MLT to achieve a complete parasitological cure in an acute murine model of *T. cruzi* infection, the positive interaction with BZ both *in vitro* and *in vivo* opens a promising alternative in drug repurposing and combinatory therapy approach for Chagas disease treatment. MLT and other synthetic alkylphospholipids arise as drug candidates for combined treated schemes and serve as chemical scaffolds for new compound entities.

## Data Availability Statement

The raw data supporting the conclusions of this article will be made available by the authors, without undue reservation.

## Ethics Statement

The animal study was reviewed and approved by Institutional Animal Care and Use Committee from the Faculty of Veterinary Sciences (University of Buenos Aires) (Protocol #2014/04).

## Author Contributions

JENG: Writing - original draft, Writing -review & editing, Investigation, Validation, Formal analysis. MMCB: Writing -review & editing, Investigation, Validation, Formal analysis. DR and MES: Investigation, Validation, Formal analysis. JA: Conceptualization, Methodology, Funding acquisition, Resources, Supervision. FG-B: Conceptualization, Methodology, Writing - review & editing, Funding acquisition, Resources, Supervision. All authors contributed to the article and approved the submitted version.

## Funding

This work was supported by Agencia Nacional de Promoción de la Investigación, el Desarrollo Tecnológico y la Innovación, Ministerio de Ciencia y Técnica, Argentina (Grant: Proyectos de Investigación Científica y Tecnológica PICTO-GLAXO 2012-035).

## Conflict of Interest

The authors declare that the research was conducted in the absence of any commercial or financial relationships that could be construed as a potential conflict of interest.

## Publisher’s Note

All claims expressed in this article are solely those of the authors and do not necessarily represent those of their affiliated organizations, or those of the publisher, the editors and the reviewers. Any product that may be evaluated in this article, or claim that may be made by its manufacturer, is not guaranteed or endorsed by the publisher.

## Footnotes

1
https://www.who.int/chagas/en/. Last access: Dec. 12, 2021.
