# Supplementary material for: Miltefosine and Benznidazole Combination Improve Anti-Trypanosoma cruzi In Vitro and In Vivo Efficacy
Source: Front Cell Infect Microbiol. 2022 Jul 5;12:855119. doi: 10.3389/fcimb.2022.855119 (PMC9294734; doi:10.3389/fcimb.2022.855119)
Supplement: Supplementary file 1 [file Image_1.pdf]

Supplementary Material

A)

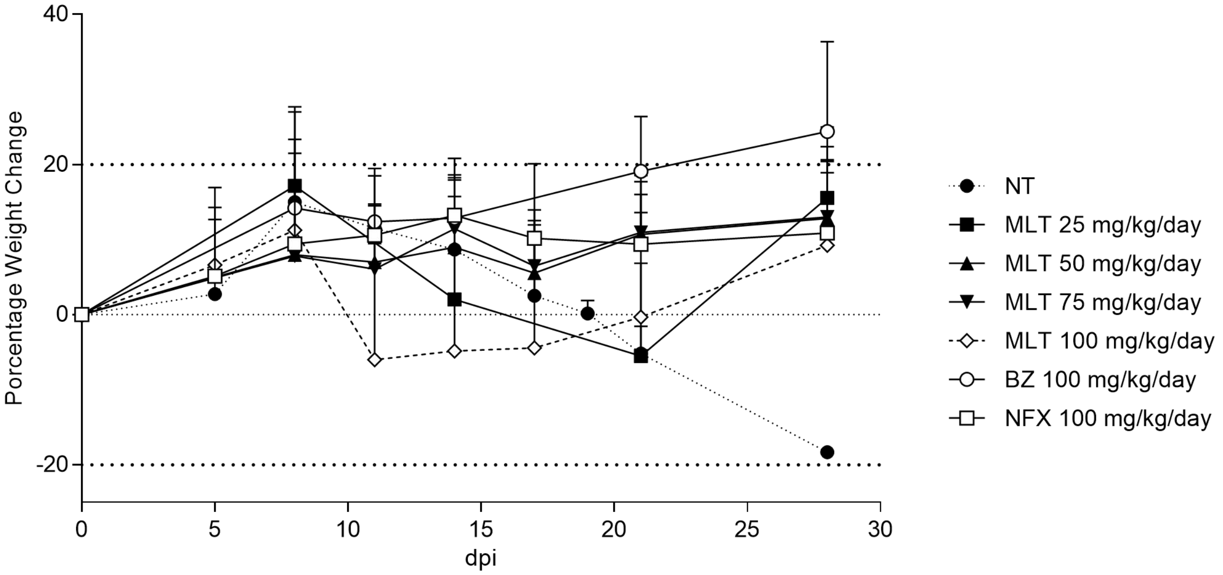

B)

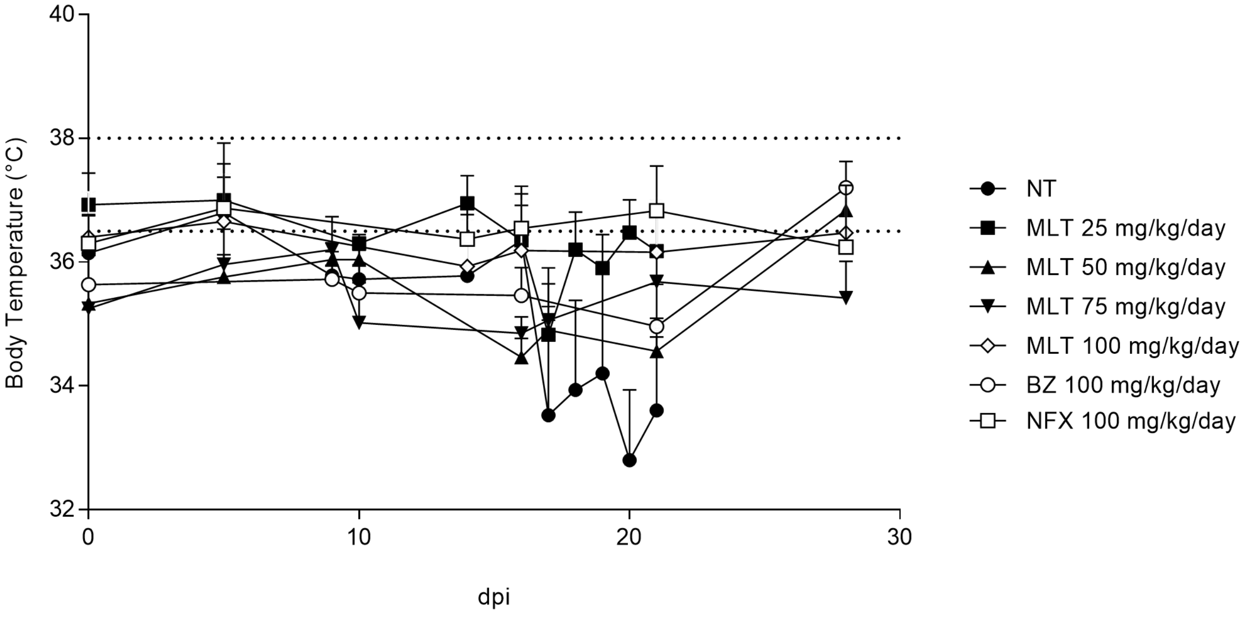

Figure S1. Effect of benznidazole (BZ), nifurtimox (NFX), or miltefosine (MLT) treatment on body weight and temperature of infected mice.

A) Evolution of percent change in body weight of infected mice measured during the acute phase of *T. cruzi* infection. Initial baseline weight (0% change) is indicated with a dashed line.

B) Body temperature of mice infected with *T. cruzi* obtained through rectal measurements. Range for normal corporal temperatures in mice (36.5-38°C) is indicated with dashed lines.

A)

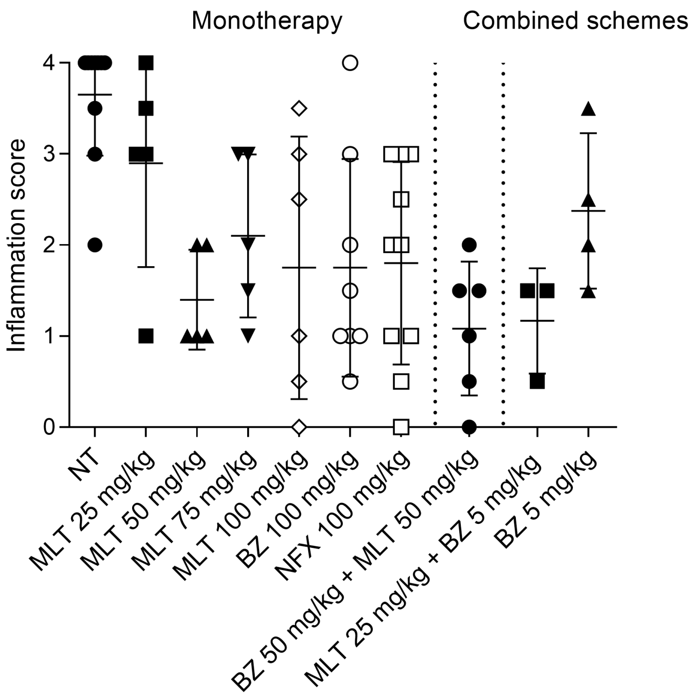

B)

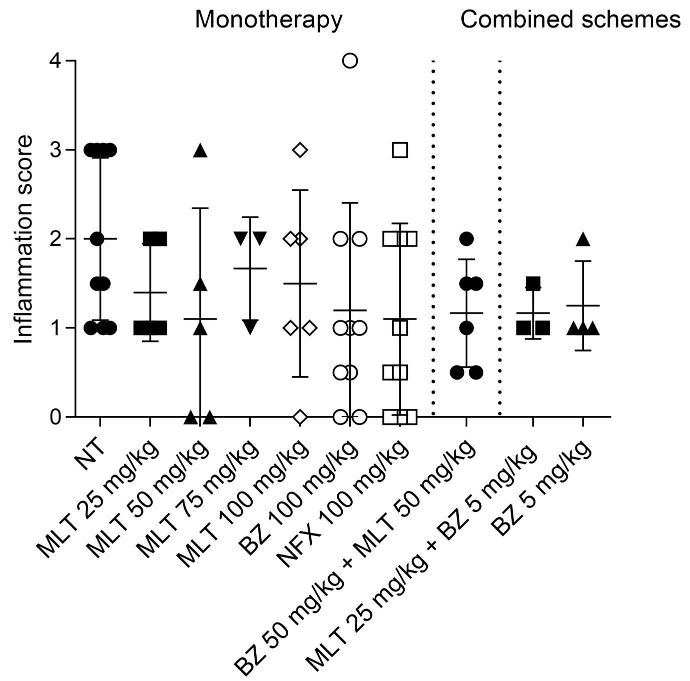

Figure S2. Inflammation score of skeletal muscle (A) and heart (B) samples from mice infected with *T. cruzi*. Increasing score indicates more severe histopathological lesions. Error bars show standard deviation.

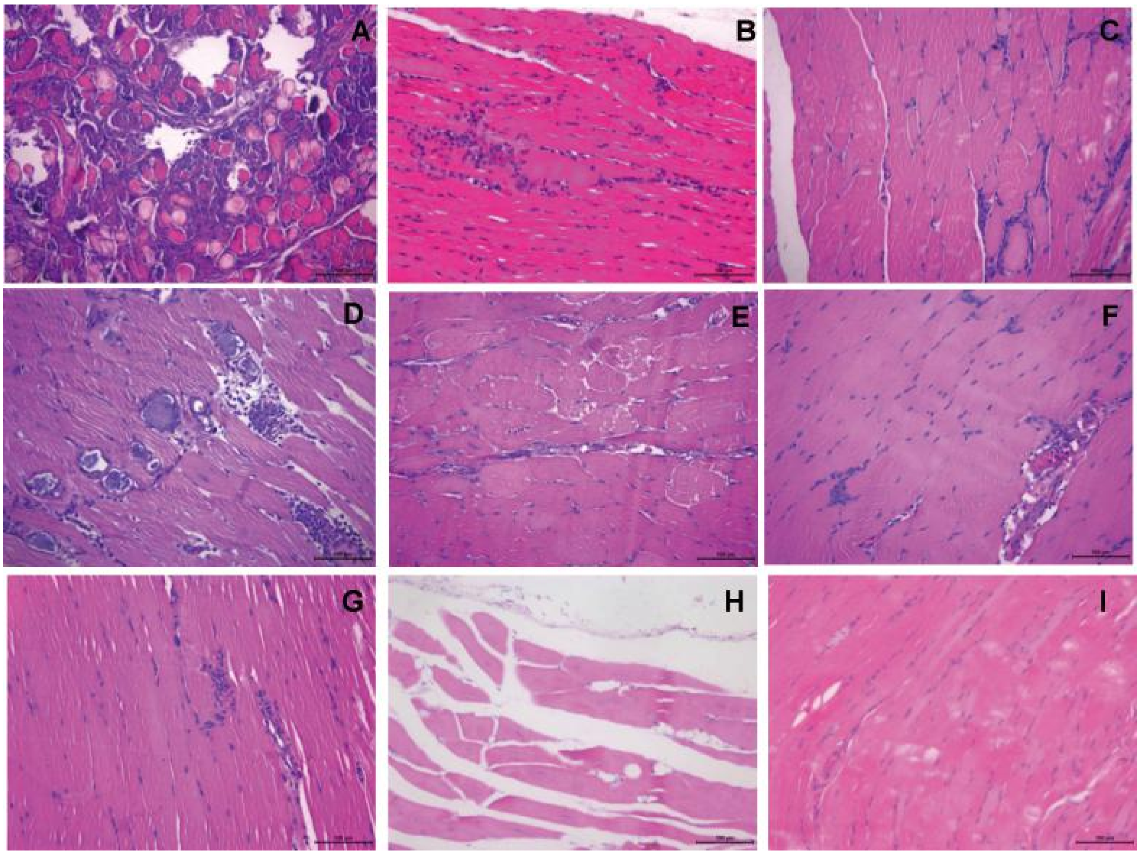

Figure S3. Microphotographs of representative samples of skeletal muscle in treatment groups. 400x magnification. Haematoxylin and eosin stain. A) Infected non-treated. B) BZ 100mg/kg/day. C) NFX 100 mg/kg/day. D) MLT 25 mg/kg/day. E) MLT 50 mg/kg/day. F) MLT 75 mg/kg/day. G) MLT 100 mg/kg/day. H) MLT 25 mg/kg/day + BZ 5 mg/kg/day. I) MLT 50 mg/kg/day + BZ 50 mg/kg/day.

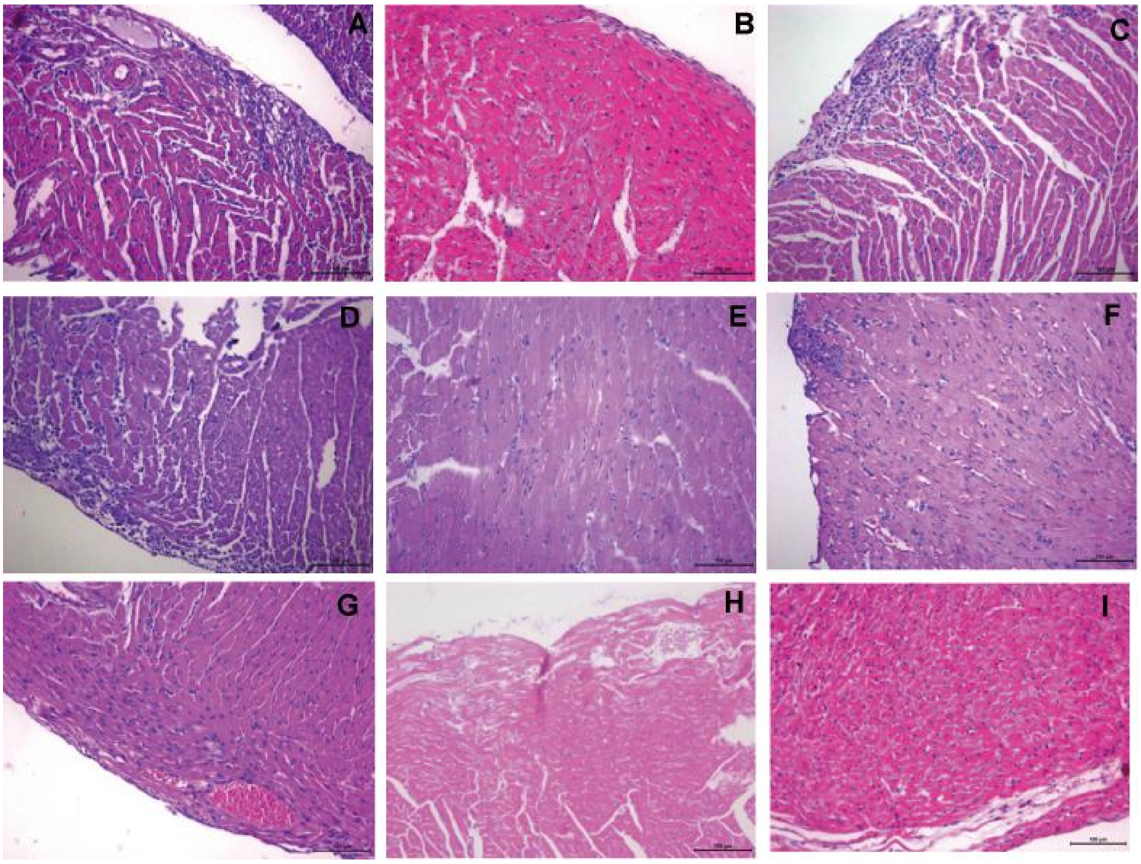

Figure S4. Microphotographs of representative samples of cardiac muscle in treatment groups. 400x magnification. Haematoxylin and eosin stain. A) Infected non-treated. B) BZ 100mg/kg/day. C) NFX 100 mg/kg/day. D) MLT 25 mg/kg/day. E) MLT 50 mg/kg/day. F) MLT 75 mg/kg/day. G) MLT 100 mg/kg/day. H) MLT 25 mg/kg/day + BZ 5 mg/kg/day. I) MLT 50 mg/kg/day + BZ 50 mg/kg/day.

A)

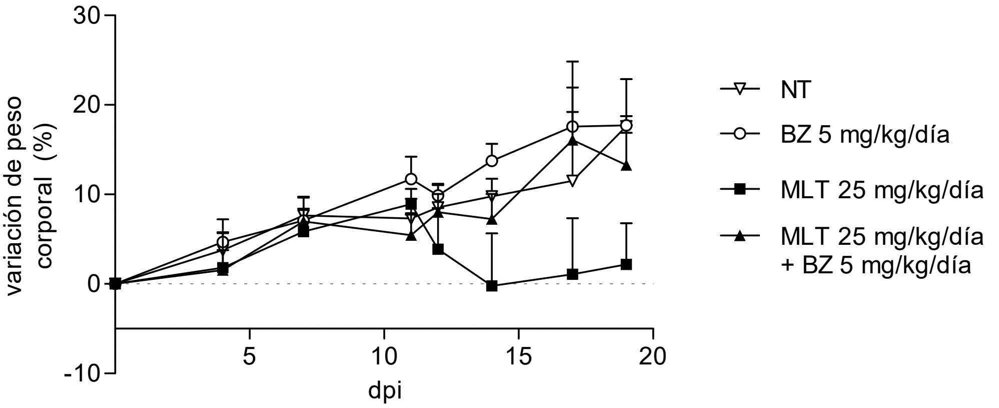

B)

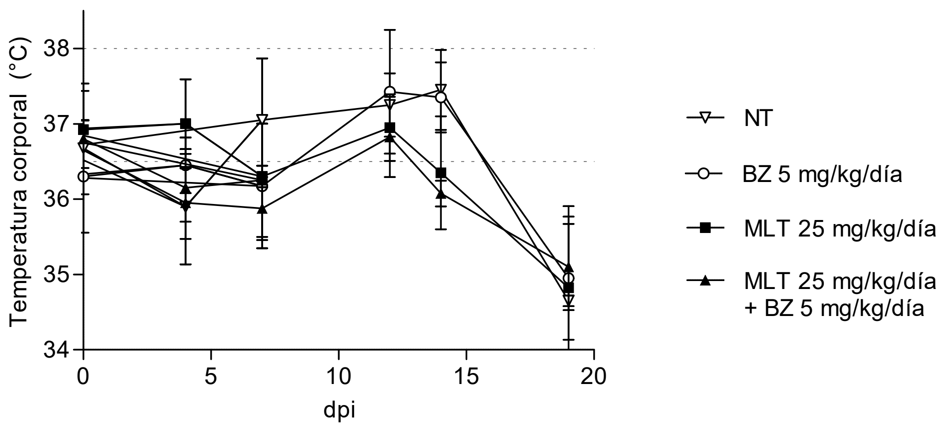

Figure S5. Effect of miltefosine (MLT) alone or combined with benznidazole (BZ) on body weight and temperature of infected mice.

A) Evolution of percent change in body weight of infected mice measured during the acute phase of *T. cruzi* infection. Initial baseline weight (0% change) is indicated with a dashed line.

B) Body temperature of mice infected with *T. cruzi* obtained through rectal measurements. Range for normal corporal temperatures in mice (36.5-38°C) is indicated with dashed lines.

A)

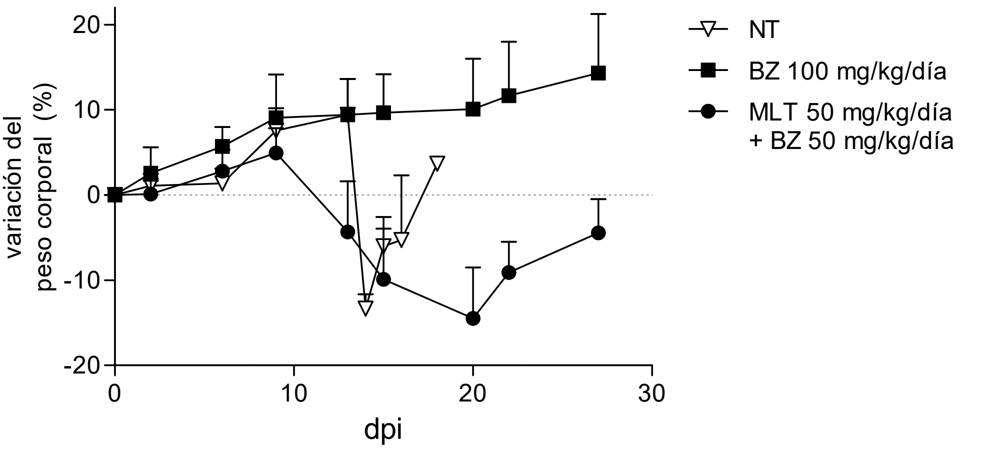

B)

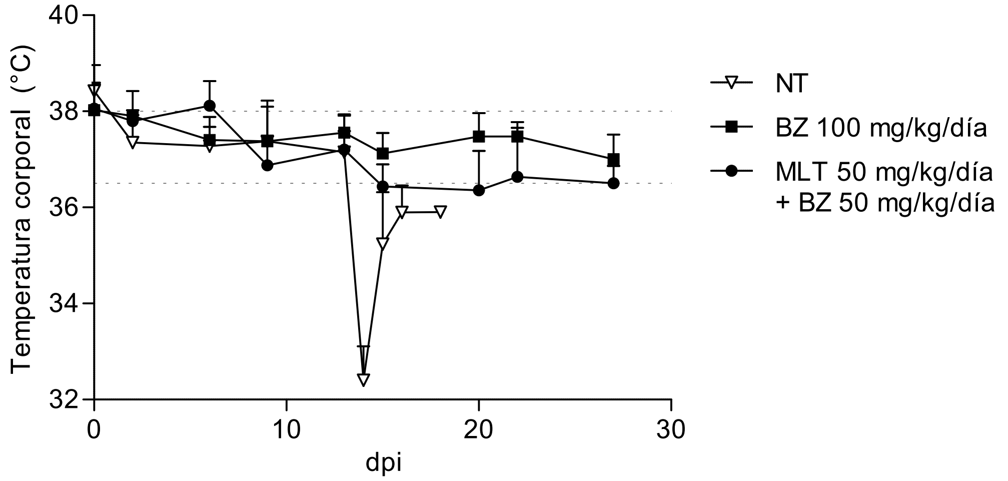

Figure S6. Effect of miltefosine (MLT) alone or combined with benznidazole (BZ) on body weight and temperature of infected mice.

A) Evolution of percent change in body weight of infected mice measured during the acute phase of *T. cruzi* infection. Initial baseline weight (0% change) is indicated with a dashed line.

B) Body temperature of mice infected with *T. cruzi* obtained through rectal measurements. Range for normal corporal temperatures in mice (36.5-38°C) is indicated with dashed lines.

A)

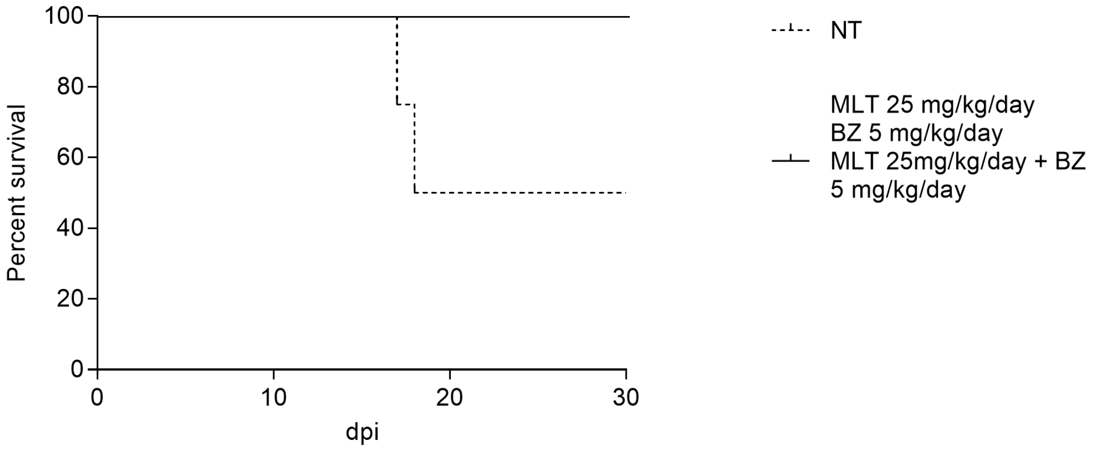

B)

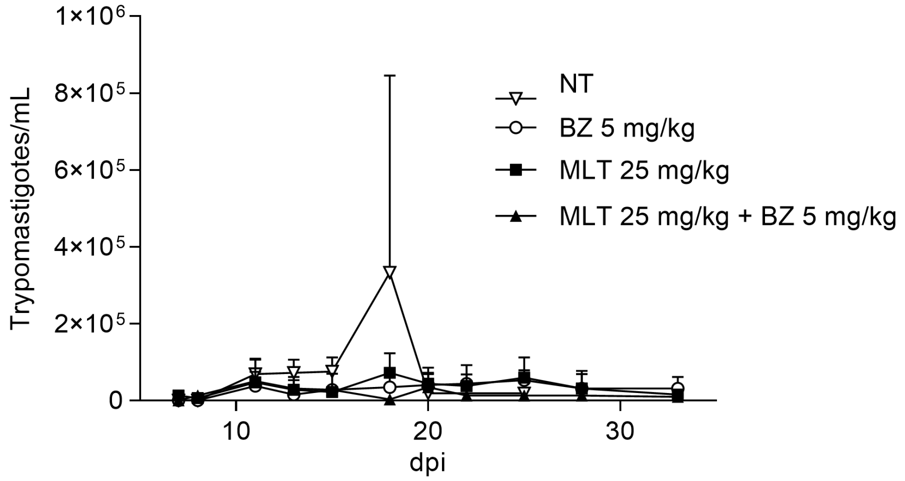

Figure S7. Effect of miltefosine alone (25 mg/kg/day) or combined with benznidazole (5 mg/kg) on (A) mortality and (B) parasitemia course in BALB/cJ mice infected with *Trypanosoma cruzi* (VD strain). Values are expressed as mean trypomastigotes/mL (± SD) in peripheral blood from experimental groups according to the days after infection (dpi).

Mice were inoculated with 500 trypomastigotes of the VD strain of *Trypanosoma cruzi,* and treatment started at parasitemia onset (8th dpi). MLT: miltefosine. BZ: benznidazole. NT: infected non-treated.

A)

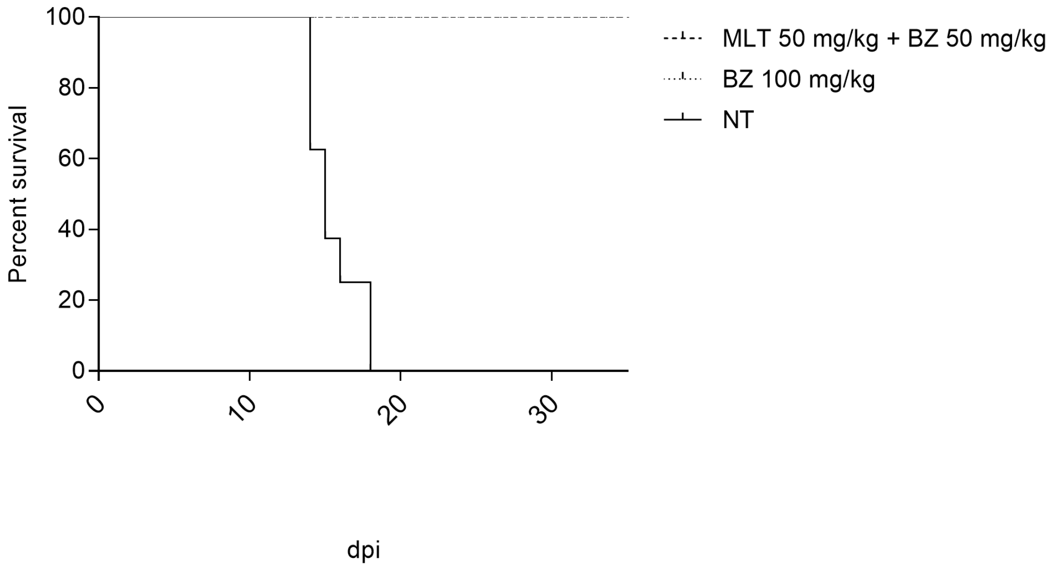

B)

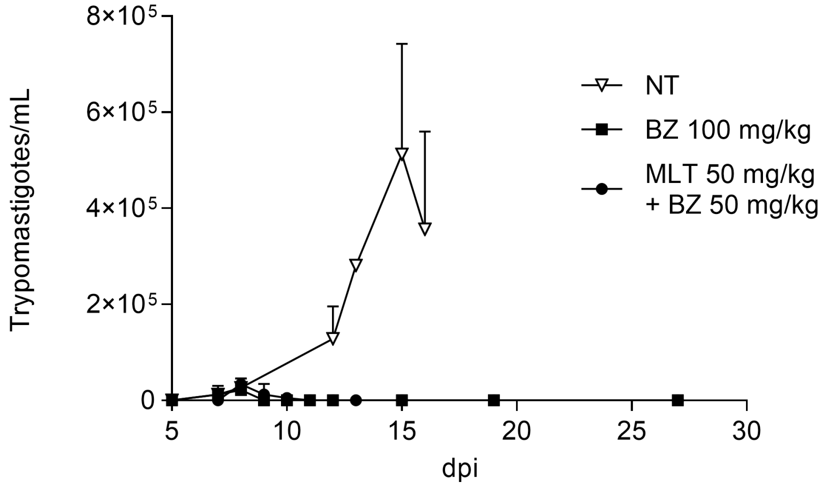

Figure S8. Effect of miltefosine alone (50 mg/kg/day) or combined with benznidazole (50 mg/kg) on (A) mortality and (B) parasitemia course in BALB/cJ mice infected with *Trypanosoma cruzi* (VD strain). Values are expressed as mean trypomastigotes/mL (± SD) in peripheral blood from experimental groups according to the days after infection (dpi).

Mice were inoculated with 500 trypomastigotes of the VD strain of *Trypanosoma cruzi,* and treatment started at parasitemia onset (8th dpi). During treatment and up to ten days post-treatment, parasitemia was evaluated by fresh blood examination (FBE) to determine parasitemia rebound. Animals with negative parasitemia were submitted to immunosuppression consisting of four doses of cyclophosphamide (CYP; 200 mg/kg; ip route), separated by one week. Parasitemia was evaluated during the CYP cycle and up to 7 days after the last dose. NT: infected non-treated. MLT: miltefosine. BZ: benznidazole.
